# The Application of Various Bark Species as a Fillers for UF Resin in Plywood Manufacturing

**DOI:** 10.3390/ma15207201

**Published:** 2022-10-15

**Authors:** Joanna Walkiewicz, Jakub Kawalerczyk, Radosław Mirski, Dorota Dziurka, Marek Wieruszewski

**Affiliations:** Department of Mechanical Wood Technology, Faculty of Forestry and Wood Technology, Poznań University of Life Sciences, Wojska Polskiego 38/42, 60-627 Poznań, Poland

**Keywords:** bark, filler, formaldehyde emission, mechanical properties, UF resin, plywood, wood-base materials

## Abstract

The aim of the presented study was to apply various bark species (birch, beech, maple, pine and spruce) as fillers for urea-formaldehyde (UF) resin in three-layer plywood manufacturing. For this purpose, all types of bark were ground and added to the adhesive mixture. The resultant plywood was subjected to investigations of the following: tensile strength, modulus of elasticity (MOE), bending strength (MOR) and formaldehyde emission. The results indicate a reduction in the tensile strength. Moreover, the lack of significant improvement in strength parameters can be explained by too high a load of the filler (20 wt%). In the case of formaldehyde emissions, a reduction was observed for birch (B-1), beech (B-2), maple (B-3) and pine bark (B-4). In addition, an increase in the emission of formaldehyde was recorded only for spruce bark.

## 1. Introduction

The adhesive mixtures used for the production of plywood usually contain some additives called fillers [1]. Their role is usually to adjust the rheological properties of the resin in order to reduce the cost of the raw material and improve the properties of the resultant panels, for example, by causing a reduction in formaldehyde emission [2,3,4].

Current trends focus on the use of most industry by-products. Following this tendency, it is worth emphasizing that the wood industry generates huge amounts of by-products, such as bark, sawdust and dust [5]. Annual global bark production is estimated to be 359,111,200 m^3^ [6], which makes it an excellent raw material that can be used in several ways. Generally, bark is used in horticulture as a mulch [7] or is considered a natural source of chemicals [8].

Due to the chemical composition of bark and the presence of numerous organic compounds, such as tannins, catechins, galocatechins, falwonoids, proanthocyaninidins, it can be used as a potential formaldehyde-scavenging filler in the production of plywood. The chemical compounds of bark, such as lignin or tannins, seem to be able to react with formaldehyde. This phenomenon may reduce the toxic effects of formaldehyde on humans by reducing hazardous emissions occurring in the indoor environments [9,10,11,12].

Formaldehyde is the simplest aldehyde, and it is widely used in the synthesis of the resins. Unfortunately, it is a harmful compound with a known carcinogenic effect. Studies on the reduction in formaldehyde emissions through the use of various types of fillers are continuously being conducted globally [1,13,14,15,16,17,18,19].

Therefore, it is worth looking at bark as a natural filler with a formaldehyde bonding ability. The use of bark can therefore contribute to a reduction in formaldehyde emissions from wood-based materials such as plywood. Currently, a lot of scientific research is focusing on the possibility of producing environmentally friendly materials. There are already existing scientific references from the literature regarding the use of larch bark to reduce formaldehyde emissions from decorative boards [20]. Moreover, Medved et al. [21] conducted studies showing that the bark of spruce and pine can be used as a substitute for wooden particles in the production of particleboard. Research on the use of the bark was also conducted by Sahin and Arslan [22], Réh et al. [23], Ružiak et al. [24]. The results of these studies also indicate that the bark species could have a significant impact on the effectiveness of reducing formaldehyde emissions.

There are many issues associated with the production of plywood that are constantly being researched. Formaldehyde emissions are a very important aspect; however, mechanical properties should also be considered as crucial for potential applications. Aydin et al. [25] reported that an amount of bark higher than 12.25% has a negative influence on formaldehyde release, thickness swelling, and mechanical strength, whereas research conducted by Mirski et al. [14] showed that the addition of oak bark at a concentration of 15% made it possible to produce plywood panels characterized by reduced formaldehyde release and improved bonding quality, which is the main goal when establishing the potential industrial application. Another important aspect is the influence of the particle size on the parameters of the produced plywood. Different sizes of particles were previously tested, and it was found that the dimensional fraction of 0.315 mm showed the best result [26]. Furthermore, the increase in bending strength and Young’s modules of elasticity was observed when 15% of beech bark was added to a urea–formaldehyde adhesive [24].

The aim of the study was to use the selected, various bark species commonly processed in Polish sawmills that were not studied before as fillers for UF resin in plywood manufacturing.

## 2. Materials and Methods

### 2.1. Materials

Plywood was produced using rotary cut birch veneer sheets with dimensions of 320 × 320 mm, moisture content of 6% ± 1%, and average thickness of 1.5 mm. An industrial UF resin with the following characteristics was used: pH 9.5 to 10.7, solids content of 64 to 69%, and gel time at 100 °C of 63 s. Ammonium nitrate (20 wt%) was introduced to the adhesive mixture as a hardener. The rye flour and bark powders differing in species were used as a fillers. For this purpose, birch (*Betula* L.), beech (*Fagus* L.), maple (*Acer* L.) pine (*Pinus* L.) and spruce (*Picea* A. Dietr.) bark particles were ground to obtain a fraction of 0.315–0.4 mm. Moisture content of added bark was ~9.0 %. The compositions and pH of adhesive mixtures are presented in Table 1.

### 2.2. Methods

The plywood panels were manufactured in the three-layer system. The adhesives mixtures were spread on the surface of veneer sheets in the amount of 170 g/m^2^. The pressing parameters, such as temperature, unit pressure and time were 120 °C, 1.4 MPa and 4 min, respectively. The moisture content of manufactured plywood was determined according to EN 322 [27]. The thickness, density and moisture content of produced plywood are given in Table 2.

The manufactured plywood were subjected to an evaluation in terms of mechanical properties, including bonding quality according to EN 314-1 (2004) [28] after soaking (10 repetitions) and modulus of elasticity (MOE) and bending strength (MOR) in a perpendicular and parallel direction to the grains of the face veneer layer (12 repetitions), according to EN 310 [29]. The results were subjected to a statistical analysis using HSD Tukey test on the significance level of α = 0.05 with Statistica 13.0 software (StatSoft Inc., Tulsa, OK, USA).

Moreover, formaldehyde emissions were also determined. For this purpose, plywood samples were tested using a flask method in accordance with PN EN 717-3 [30] (2 repetitions).

SEM pictures were taken with a Hitachi SU 3500 Electron Microscope (Hitachi, Japan) under high-vacuum conditions. The plywood samples were covered with gold. Au sputtering was performed with a Cressington Sputter Coater 108 auto sputtering machine (Ted Pella, Redding, CA, USA).

## 3. Results

### 3.1. Tensile Strength

**Figure 1:**
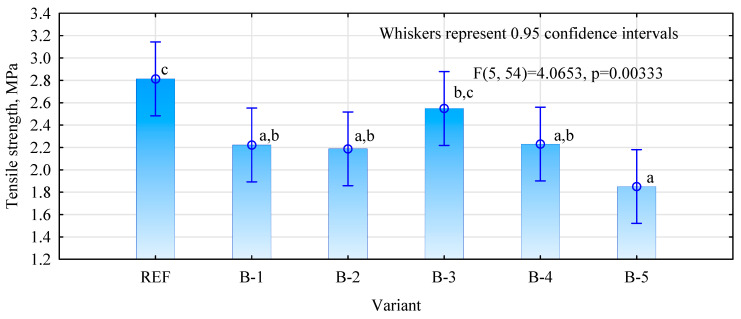
The changes in tensile strength of the produced plywood with bark: (B-1), beech (B-2), maple (B-3) and pine bark (B-4), spruce(B-5) and reference sample (REF). The lowercase letters means homogeneous groups.

The changes in tensile strength depending on the resin formulation are presented in Figure 1. As can be seen, all variants (control and plywood with bark particles) fulfilled the standard requirements for tensile strength (values exceeded 1 N/mm^2^). Moreover, a statistical analysis was carried out and homogeneous groups were distinguished. They are marked with letters above the bars. The highest values were obtained for both the control variant and maple-bark-containing one. Between them, no statistically significant differences were observed. This means that the addition of maple bark to the UF resin gave comparable results with the control variant. The lowest tensile strength value was shown by variant B-5; however, there are no statistical differences between this variant and variants B-1, B-2 and B-4. Similar results, where a reduction in tensile strength was observed due to the addition of the bark, were presented by Aydin et al. [25].

This phenomenon can be explained by the pH of the environment and its effect on the adhesive curing process. Elbadawi et al. [31] reported that the curing rates of formaldehyde-based resins are strongly dependent on the pH of the curing environment. According to Xing et al. [32], when the pH rises above 7 the reactivity of adhesive considerably slows down and it affects bonding strength. On the other hand, if the pH is too low then a pre-curing process may occur. They also observed that as the content of tannins increased (which are included in bark composition), the mechanical properties of the produced panels decreased. Furthermore, the similar results were also observed by Nemli et al. [33]. The phenomenon of lowering tensile strength can be explained by the presence of tannins, which affect the resin curing process. If there is a too major a decrease in the pH, it can lead to the pre-curing of the adhesives before pressing, and thus it can cause a deterioration in the properties of boards [25,31].

### 3.2. Modulus of Elasticity (MOE) and Bending Strength (MOR)

**Figure 2:**
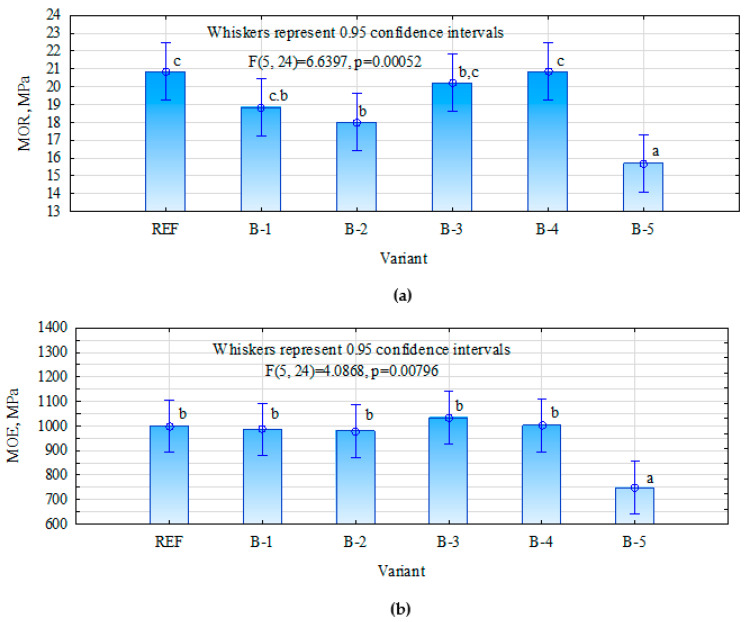
The results of MOR (**a**) and MOE (**b**) tests in perpendicular direction. The lowercase letters means homogeneous groups.

**Figure 3:**
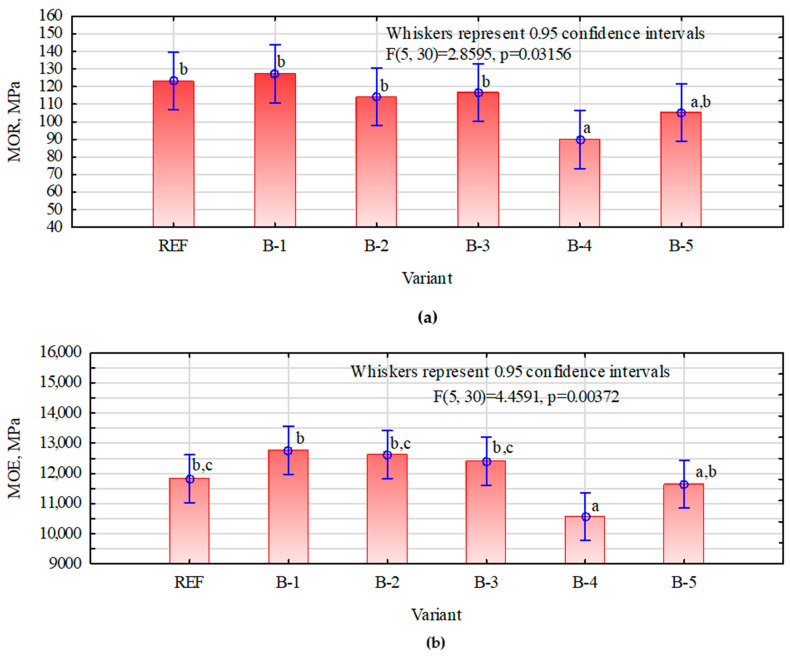
The results of MOR (**a**) and MOE (**b**) tests in longitudinal direction. The lowercase letters means homogeneous groups.

The MOR and MOE parameters were tested in two directions (longitudinal and perpendicular) (Figure 2 and Figure 3). In the case of the perpendicular direction, the statistically significant differences were observed between the individual variants (Figure 2). The results of the B-1, B-3 and B-4 variants, assuming the use of birch, maple and pine bark as fillers, are similar to the control sample (no statistically significant differences were observed). In the case of the B-5 samples, where spruce bark was applied, a significant decrease in MOR was observed when compared to the flour-filled samples. In the case of MOE, studies showed that, in the case of the variants REF, B-1, B-2, B-3 and B-4, no statistically significant differences were observed. However, as in the case of MOR, variant B-5 showed a significant deterioration.

The lack of significant improvement in strength parameters—and in the case of the B-5 variant, their significant reduction—can be explained by too high a proportion of the filler used. According to the literature, an addition of more than 15% significantly increases the viscosity of the resin [23,24,34]. Réh et al. [35] also observed that the addition of 20% (based on the dry weight of the resin) of bark particles increases the viscosity of the resin. This can result in an uneven application of glue.

Another important aspect is the presence of tannins and lignin in the bark, which influences the properties of the resin. Various explanations can be found in the literature, which still remain inconclusive. On the one hand, a high proportion of these compounds can lead to an improvement in mechanical properties [35]. On the other hand, in some cases, a high proportion of these compounds affects their reactivity, which leads to a rapid increase in viscosity, which in turn translates into a short resin pot life and affects intermolecular cross-linking [36,37]. The observations of Nemli et al. [33] confirm that, in this case, the MOE results can be lowered.

Figure 3 presents the results of MOE and MOR in the longitudinal direction. It was observed that MOR parameters for the variants REF, B-1, B-2 and B-3 were at a similar level, and there were no statistically significant differences. Only in the case of B-4 and B-5 variants was there a slight decrease, which may have been observed due to the decreased veneer quality. In the case of MOE, the differences were noticeable, and statistically significant differences were observed. As in the case of the MOR, the MOE was the lowest for the B-4 variant.

### 3.3. Formaldehyde Emission

**Figure 4:**
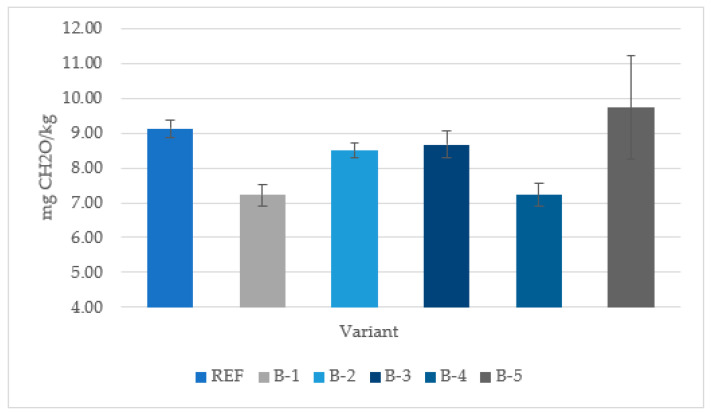
Formaldehyde emission in three-layer plywood panels produced with UF resin.

Figure 4 presents the results of the formaldehyde emission of the laboratory-manufactured plywood panels bonded with UF resin and the addition of different wood bark species as fillers. The results of experiment clearly indicate that the replacement of rye flour with birch (B-1), beech (B-2), maple (B-3) and pine (B-4) bark led to a decrease in formaldehyde emissions compared with the reference samples (REF). The reduction in formaldehyde emissions can be explained by the presence of both lignin and tannins in bark. Van Der Klashorst and Strauss [38] reported that lignin is able to react with formaldehyde in an acidic medium. Moreover, tannins are characterized by their phenolic nature, and condensed polyflavonoid tannins are able to react with HCHO [39,40]. Only in the case of spruce bark (B-5), an increase in the emission of formaldehyde was recorded. This can be explained by the weakening of the bonding quality, which was confirmed by the results for the mechanical properties. According to Hogger et al. [41], the hindrance in polymer network formation and a deterioration in bonding quality can result in an increase in formaldehyde emission from UF resin-bonded plywood.

### 3.4. SEM Analysis

**Figure 5:**
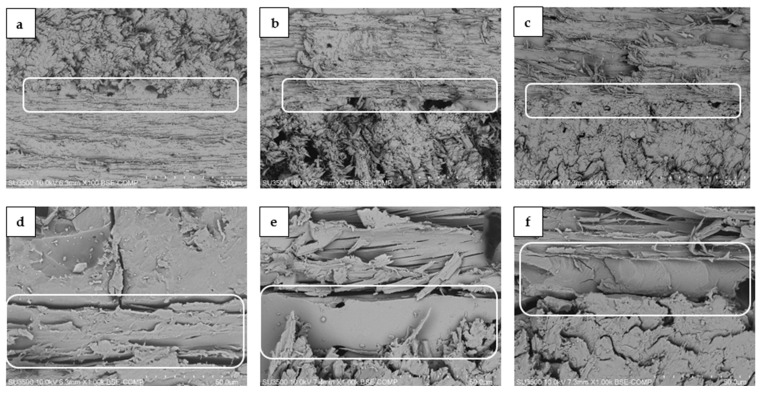
SEM photos of cross-section plywood: (**a**,**d**)—REF; (**b**,**e**)—B-1; (**c**,**f**)—B-5 samples. The bond lines are marked with white frames box.

The cross-sections of plywood characterized with SEM at different magnifications of ×100 and ×1000 are presented at Figure 5. Compared with REF resin plywood (Figure 5a,d), the glue line of B-1 and B-5 adhesive plywood was characterized by larger free spaces (Figure 5b,c,e,f). This could be caused by the fact that bark fillers contained adhesive B-1, and B-5 was characterized by larger particle size. However, these changes affected the mechanical parameters (MOR and MOE in perpendicular direction) and formaldehyde emissions only in the case of the B-5 sample when compared to the REF sample.

## 4. Conclusions

In the case of maple bark, there were no statistically significant differences compared to the reference sample. In other cases, the reduction in the tensile strength was observed after the addition of the bark when compared to the reference variant. The phenomenon of lowering tensile strength can be explained by the chemical composition of bark, which could affect the resin curing process. Moreover, when the pH is considerably lowered, the pre-curing process could occur and consequently affect the bond lines strength.

The lack of significant improvement in strength parameters (MOR, MOE) can likely be explained by too high a proportion of filler (20 wt%).

The results of the experiment clearly indicate that the replacement rye flour with birch (B-1), beech (B-2), maple (B-3) and pine (B-4) bark led to a decrease in formaldehyde emissions. The observed reduction in emissions can be explained by the high lignin and tannins content. Only in the case of spruce bark (B-5), an increase in the emissions of formaldehyde was recorded. These results can be explained by the noted deterioration in the bonding quality, which is in agreement with the results of mechanical properties.

The most important conclusion is that the use of 20% (wt) bark powder is too much, and a lower load of filler should be used in the future studies.

## Figures and Tables

**Table 1:** Compositions of adhesive mixtures with various type of bark particle.

Variant Label	Type of Filler	Quantity (pwb Per 100 pwb of Solid Resin)		pH
		Filler	Hardener Solution	
REF	Rye flour	20	2	6.88
B-1	Birch bark	20	2	6.57
B-2	Beech bark	20	2	6.50
B-3	Maple bark	20	2	6.89
B-4	Pine bark	20	2	6.21
B-5	Spruce bark	20	2	6.26

Note: pbw means parts by weight.

**Table 2:** Parameters of manufactured plywood.

Variant Label	Density [kg/m^3^]	Moisture Content [%]	Thickness [mm]
REF	626	5.0	3.98
B-1	689	5.1	4.04
B-2	679	5.2	4.16
B-3	698	4.8	3.95
B-4	643	4.6	3.94
B-5	602	5.2	3.92

## Data Availability

Not applicable.
